# Seismic Performance Evaluation According to HSS and CFST Columns of 3D Frame Buildings with Rubber Friction Bearing (RFB)

**DOI:** 10.3390/ma15041281

**Published:** 2022-02-09

**Authors:** Young-chan Kim, Hasan Shahriyer, Jong-wan Hu

**Affiliations:** 1 Incheon Disaster Prevention Research Center, Incheon National University, Incheon 22012, Korea; channy0409@inu.ac.kr; 2 Department of Civil and Environmental Engineering, Incheon National University, Incheon 22012, Korea; hasanshahriyer@inu.ac.kr

**Keywords:** 3D structure, HSS, CFST, steel BRBs, rubber friction bearing, basement frame

## Abstract

This study has been conducted to observe nonlinear time history analysis of a 3D-office building frame where performance has been examined in the presence of base isolation and a bracing system. This steel structure has an underground story surrounded by stiff well-graded sand and is assumed to be located in an intense seismic area. The static and dynamic experimental performance of a Rubber Friction Bearing (RFB) has been considered, and an equivalent numerical model has been used in finite element software, which provides a satisfactory relationship between experimental and numerical prediction. The results show that the story drift and post-earthquake damage of the frame reduced significantly due to the presence of RFB devices. These isolators are most effective in moderate earthquakes. The presence of a minimum number of Steel Buckling Restrained Braces (BRBs) systems improve structural performance under moderate and strong ground motions by reducing story drift and residual damage. Hollow Steel Section (HSS) and Concrete-Filled Steel Tube (CFST) sections have been used in the simulation process, and it was found that the HSS system is susceptible to damage even if both seismic protection systems have been considered. The findings provide important conclusions to select suitable seismic protection for this type of structure, which is limited by simulation study due to the absence of experimental observation.

## 1. Introduction

In comparison to other natural disasters, earthquakes (EQs) are the most common and do not provide significant signs prior to this unpredictable event. EQs cause damage to the properties and loss of life every year around the world. In the United States of America (USA), the financial damage is approximately 6.1 billion USD per year [1]. Life safety and collapse preventions are the two main design philosophies for the past seismic design technology, which might have been well adopted by ensuring no life loss; preventing failure of the structure [2,3] requires upgrading to a “Performance-Based Design (PBD)” framework [4,5,6]. PBD ensures the safety of the structural and nonstructural components as well as decreases the vulnerability of the building by reducing the EQ demand within the system itself, which results in a high-performance building.

Representatively, during the 1994 Northridge and the 1995 Kobe earthquakes, significant amounts of brittle fracture occurred between the beam–column weld joints of moment-resisting frames constructed according to existing design philosophies [7]. To improve the seismic performance, researchers [7,8,9,10] came up with different kinds of bracing systems, namely Steel Buckling-Restrained Braces (BRBs) and Concentrically Braced Frames (CBFs) equipped with complex energy dissipating devices [11,12,13], which show promising results and can be used as an effective tool to provide high-level sustainable performance by dissipating excessive nonstructural damage, resulting from story drift. These bracing systems can also be considered as a long-term economical solution due to their low manufacturing, installation, and repairing costs. By replacing a newer one of this deformed bracing system, the mainframe can moderately recover to its original form, which makes braced frames superior to other structures [9].

In addition, to improve the seismic performance of structures, the seismic base isolator has been used for more than a century due to its effectiveness and economic benefits, which act as an insulator between the fixed ground and the structure by consuming the displacement demand created by the seismic events [14,15]. Different kinds of base isolation have been developed and can be broadly classified into two groups: elastomeric and sliding types [16,17]. The performance of an elastomeric bearing, which is mainly composed of synthetic or natural rubber, has been enhanced by using steel plates. Furthermore, to overcome the limitations of elastomeric bearings in small intensity EQs, elastomeric bearings using high-damping rubber and lead–rubber bearings (LRB) with a lead core in the center of the bearing were developed [18,19]. The Sliding Bearing surface has been designed to limit the force in the horizontal directions where different geometry or spring has been used with Frictional Pendulum (FP) [20]. Recently, the Multi-Spherical Frictional Pendulum bearing device has been widely installed in various countries due to various advantages such as damping property and stiffness [20]. Furthermore, researchers use soft computational techniques [21] and wavelet variance tools [22] to investigate the performance of base-isolated, braced, and uncontrolled building frames where they not only justify the results but also suggested that those techniques and tools can be effective and reliable to evaluate the response of structures under seismic loads.

In addition to this, due to urbanization, most of the population nowadays is highly concentrated in metropolitan regions, which will surely increase over time [23]. Basements are more common in medium to high-rise buildings these days, which not only provide additional floor area for vehicles, storage, and shopping space but also reduce net bearing pressure [24]. Therefore, some researchers are considering 3D frame structures with the interaction of the basement and the ground. It was noted that soil structural interaction (SSI) not only increases the dynamic demand but also increases the forces and deformations with the building height. This scenario is also affected by the number of underground stories and can lead to unsafe designs [25]. Not only that, ignoring the SSI influence may underestimate the damage that may appear after EQs.

In this study, the active and passive force displacement behavior of soil has been considered in a simplified way to observe the frame response under moderate and strong ground motion, which is most of the time ignored in order to avoid complex calculation. This six-story building frame has been designed by following the code specification given by the ASCE 7-16 [26] and AISC-LRFD manual [27]. Story drift is considered as a basic design parameter to assess the performance of the building structure [9,28,29,30]. Residual displacement is well-defined by Kawashima [31] from the 1995 Kobe earthquake, which is often considered as a key performance-based limit design factor that has been used here to define the damage control level [9,32]. Two different column systems have been used in the simulation process while considering that one building frame has an HSS column and another is composed of CFST. Rubber Friction Bearings (RFB) developed by the research team have two distinctive force–displacement relationships depending on the elastic modulus of the rubber material. A controlled building frame response by using these bearing devices has been documented here. To improve the performance of the building frame, a minimum number of well-established Steel Buckling Restrained Brace (BRB) [7,9] systems are also accounted for in this numerical observation. The key motivation of this research is the performance evaluation of RFB devices that have low lateral stiffness and their influence over the embedded frame system. Damage minimization in terms of the residual story drift along with soil spring response, base isolator response, and base shear force are also shown in this study. 

In this study, the design and experimental results of RFB conducted in previous studies were first summarized. An analysis model simulating RFB was completed. In addition, the design was performed, and the analysis model was completed for the structure with an RFB having two columns surrounded by the ground. Finally, seismic performance evaluation was performed by analyzing the results obtained by performing time history analysis on the analysis model.

## 2. Rubber Friction Bearing (RFB)

### 2.1. Physical Design of RFB

**Figure 1:**
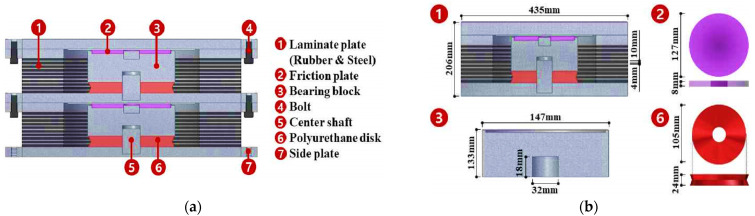
Basic Rubber Friction Bearing (RFB) device [34]: (**a**) RFB components; (**b**) Details of the design of each stack of RFB.

RFB devices and their components that have been developed by the research team are shown in Figure 1a. General elastomeric rubber and engineering plastics (EP) friction material have been used to fabricate the RFB. It has a cylindrical outer shell and inner core parts. This device uses frictional plate and polyurethane rubber at the top and bottom of the bearing block inside the device, which dissipates most of the vibration, as shown in Figure 1b [33]. The rest of the energy is dissipated by the outer cylindrical part consisting of rubber and a steel plate, which is similar to the conventional elastomeric bearing. The inner core damps the vibration due to the presence of a polyurethane disk and a frictional plate, which also helps the outer laminated plate to minimize the residual displacement. This RFB exhibits the characteristics of the combined frictional and stiffness behavior.

(1) $$k_{h}=\frac{\mathrm{GA}}{{(t}_{R}{\times n}_{\mathrm{rub}})/n}$$

(2) $$k_{V}=\frac{{A\times E\times (1+\mathrm{SF}}^{2})}{{n\times t}_{R}}$$

**Figure 2:**
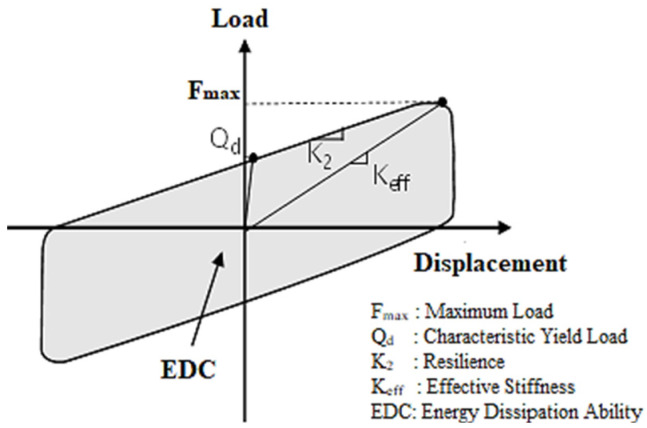
Behavioral characteristics of the RFB device.

A representative force–displacement behavior is shown in Figure 2. Depending on the shear modulus of rubber presented on BS EN 1337 [35], RFB devices are categorized into two groups: 0.45 MPa and 0.90 MPa. Table 1 shows the mechanical properties of rubber friction-bearing devices. An allowable displacement limit of ±150 mm has been considered. Horizontal stiffness is the total stiffness that the rubber layer receives at the design stage, which can be determined by Equation (1). Equation (2) calculates the vertical stiffness. In the equation, G is the shear modulus, A is the cross-sectional area, t_R_ is the height of a single rubber layer, n_rub_ is the number of rubber layers, n is the number of layers, and SF stands for shape factor.

### 2.2. Analytical Modeling of RFB

**Figure 3:**
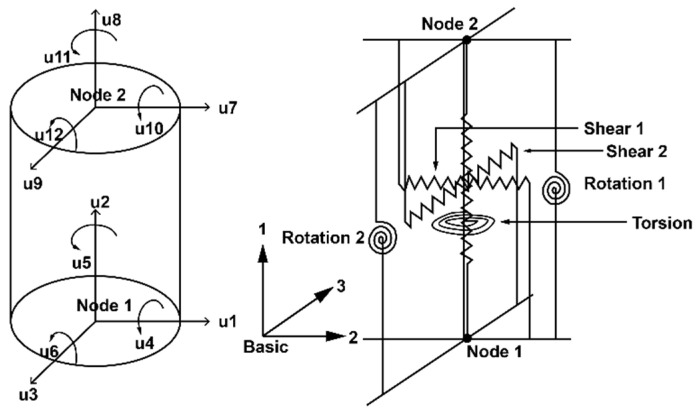
“LeadRubberX” DOF and spring systems [36].

To calibrate the force–displacement behavior of the RFB device, the “LeadRubberX” bearing element developed by M Kumar which is available in the OpenSEES platform has been considered [36]. This model consists of two nodes and 12 degrees of freedom discrete elements, as shown in Figure 3. Six separate spring systems connect these two nodes, which characterize the mechanical response of the bearing system in six directions. To match the experimental quasi-static and dynamic response of the base isolator with the numerical model, following the parameter of the “LeadRubberX” element has been considered, which is given in Table 2, and a default value has been assigned to define the remaining parameters in the simulation process. The distance between the nodes is 372 mm, which is equivalent to the height of the physical model.

### 2.3. Verification of RFB Devices

The physical test procedure, equipment specification, and results have been documented in this section. Mostly, the U.S.A and Japan are actively developing seismic isolation systems by using a standard design and verification process [37]. Recently, the number of studies on the development of different seismic base isolator systems has increased in South Korea. A common standard practice in the design and verification process of base isolation systems has been adopted for this study, which is under draft stage [38,39]. 

**Figure 4:**
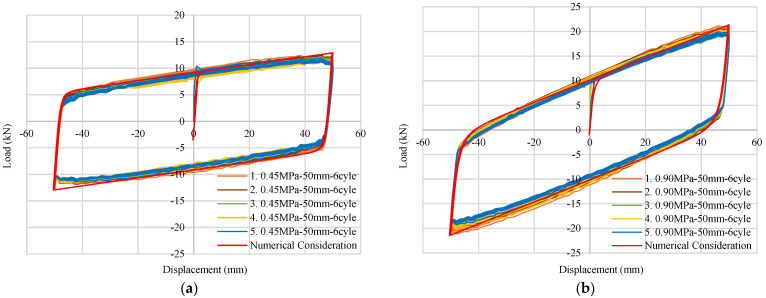
Experimental and numerical quasi-static test data of RFB devices: (**a**) Force–displacement relation of 0.45MPa RFB; (**b**) Force–displacement relation of 0.90 MPa RFB.

To perform a quasi-static loading test, the Rubber Frictional Bearing devices have been placed in a hydraulic actuator. A 50 kN load in the horizontal direction along with a 100 mm/s loading rate have been used in the hydraulic actuator. The test specimen has been divided into two groups, depending on the rubber elastic modulus (0.45 MPa and 0.90 MPa). Five specimens from each group have been used in the static cycle test process. After applying a vertical load of around 60 kN, six consecutive quasi-static horizontal loading cycles have been applied over each specimen. The maximum horizontal displacement has been considered to be ±50 mm with a speed of 100 mm/s. Test results including the numerical consideration of force–displacement behavior have been shown in Figure 4. Figure 4a,b denote the results of the 0.45 MPa and 0.90 MPa RFB devices, respectively.

For the dynamic test of the base isolators under dynamic loading condition, a 5 m by 5 m shaking table has been selected, which has three degrees of freedom (translation in the horizontal axis and rotation along the vertical axis) and a maximum load capacity of 60,000 kg. This table can generate a maximum acceleration of 3 g within a frequency range of 0.1 to 60 Hz. Four identical base isolators have been used on the shaking table in a way to fix the loaded plate. Five steel plates weighing a total of 24 tons are stacked on top of each other, creating a total load of 235.36 kN on the base isolators. To perform the dynamic test, six bi-directional earthquakes have been selected, among which three are artificial and the rest of them are the El Centro, James RD, and San Francisco EQs.

**Figure 5:**
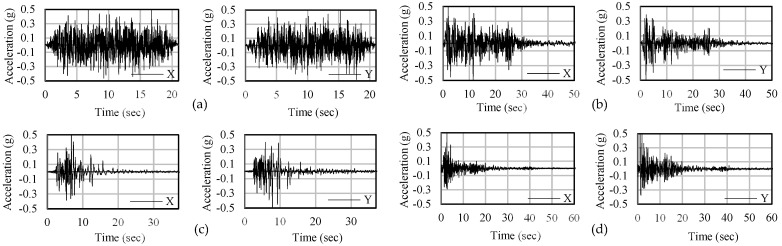
Representative bi–directional earthquake (0.5 g) data: (**a**) Artificial, (**b**) El Centro, (**c**) James RD, and (**d**) San Francisco.

**Figure 6:**
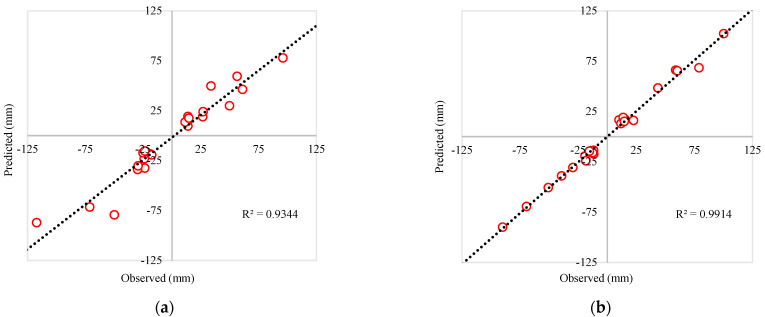
Dynamic displacement response of Base Isolator systems: (**a**) 0.45 MPa Base Isolator Dynamic Response; (**b**) 0.90 MPa Base Isolator Dynamic Response.

Earthquakes are scaled down to a peak ground acceleration of 0.5 g. Representative single artificial and above-mentioned earthquake data are shown in Figure 5, where the artificial earthquake has a duration of 22.5 s and the El Centro, James RD, San Francisco earthquakes have a duration of 54, 38, and 62 s, respectively. Detailed design consideration of the earthquakes has also been discussed afterward. Figure 6a,b display the coefficient of determination of peak dynamic displacement of two different Rubber Friction Bearing devices. In both cases, “R squared” values close to 1 were found, which were within the acceptable range.

## 3. Soil–Structural Interaction 

### 3.1. Soil–Wall Boundary

**Figure 7:**
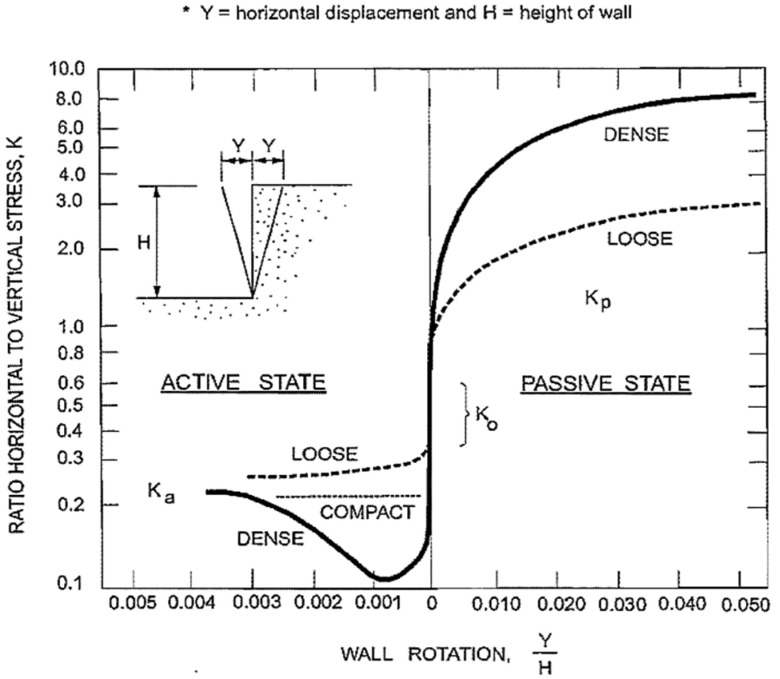
Force–deformation relationship in cohesionless soil.

**Figure 8:**
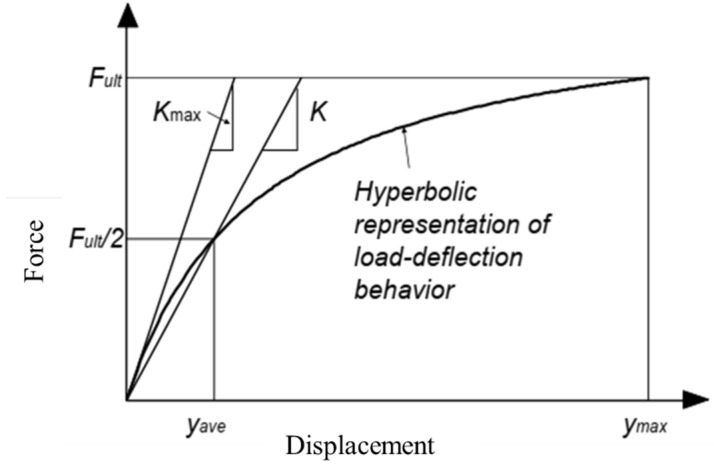
Hyperbolic force–displacement behavior.

Coulomb, Rankine, and Log Spiral approaches [40,41] are generally used simplified assumptions to calculate passive pressure, which requires precise values of unit weight of soil (γ), friction angle of soil (φ), cohesion (c), the supported height of soil (H), soil–wall boundary friction (δ), and other geometric properties of the embankment wall and backfill. The above-mentioned theories do not provide any force–displacement relationship, which plays a major part in practical cases such as abutment deflection of bridges [42,43,44], horizontal resistance of shallow foundation, and pile cap [45,46,47]. The Canadian Foundation Engineering manual developed by the Canadian Geotechnical Society [48] provides figures showing the relation between soil strain and horizontal stress to achieve active and passive earth pressure conditions, which have been shown in Figure 7. The hyperbolic model, as shown in Figure 8, provides a good representation of the load–displacement behavior of experimental data. Shamsabadi et al. (2007) model is based on the secant stiffness whereas Duncan and Mokwa (2001) provides a hyperbolic model, which is defined by the initial stiffness (Kmax) [43,49].

(3) $$F\left(y\right)=\frac{F_{\mathrm{ult}}\left({2\mathrm{Ky}}_{\max}{-F}_{\mathrm{ult}}\right)y}{F_{\mathrm{ult}}y_{\max}+2\left({\mathrm{Ky}}_{\max}{-F}_{\mathrm{ult}}\right)y}$$

(4) $$F\left(y\right)=\frac{y}{\frac{1}{K_{\max}}{+R}_{f}\frac{y}{F_{\mathrm{ult}}}}$$

Under earthquake loading, the backfill soil and surrounded wall response is highly complex and difficult to predict; also, internal force and earthquake motion increase the demand on the building system. For the design and analysis of a retaining structure under seismic excitation, two of the most popular theoretical procedures have been used to calculate the dynamic earth pressure. In Mononobe–Okabe [50,51] equations, the wall has been permitted to achieve sufficient displacement under active and passive conditions, which are used for “yielding” wall conditions. The second one is an elastic method proposed by Wood (1973), which argues that “non-yielding” walls—for example, basement walls—that are rigid cannot achieve the active or passive condition [52].

### 3.2. Simulation Consideration of Soil Domain

Accurate modeling of passive force–displacement earth pressure resistance at the retaining wall, abutment, and pile caps can provide a more economical and realistic safer design which nowadays is limited by a smaller number of bilinear design models and tests data. In most of the cases, it does not consider the nonlinear performance, inertial effect of the building structures, and backfills, which may have a significant influence over dynamic shaking [53]. By using zero-length elements [54] at the surrounding of the three-dimensional building frame, the soil wall and building frame have been separated. The Hyperbolic Gap Material (HGM) model has been used to characterize the passive force–displacement in dynamic basement wall simulations [55].

As discussed earlier, the hyperbolic model shows a good representation of the load–displacement behavior of the experimental data; the model of Shamsabadi et al. (2007) has been designed based on a secant stiffness K, which can be described by Equation (3). In Equation (3), F represents the resisting force, y represents the horizontal displacement, F_ult_ is the maximum passive resistance, and K is the stiffness at F_ult_/2 [43].

**Figure 9:**
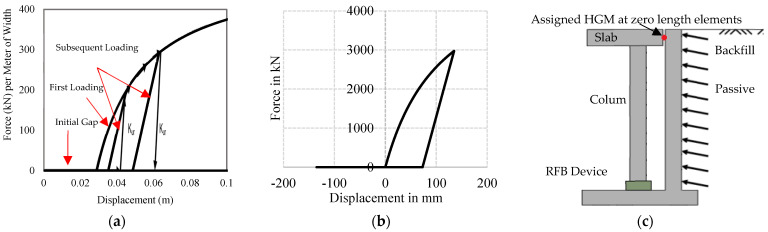
Behavior characteristics and location of the soil spring: (**a**) Behavioral characteristics of HGM; (**b**) Static HGM response; (**c**) Schematic view of the ground.

On the other hand, the hyperbolic model of Duncan and Mokwa (2001) is defined by the initial stiffness (K_max_) by using the following Equation (4) where R_f_ represents the failure ratio [49]. The typical behavior of HGM material is shown in Figure 9a, where the initial stiffness has been denoted by K_max_. Unloading–reloading has been defined by K_ur_, failure ratio is represented by R_f_, the ultimate passive resistance is F_ult_, and the initial gap has been considered by the gap [54]. For simulation purposes, the gap has been considered negligible, as shown in Figure 9b.

Dense well-graded silt sand has been considered in the simulation process in which the initial stiffness (K_max_) is 48,000 kN/m/m, the unloading reloading stiffness (K_ur_) is 48,000 kN/m/m, the failure ratio (R_f_) is 0.1, the ultimate (maximum) passive resistance (F_ult_) is 550 kN/m, and an initial gap of 0.01 mm has been assigned in the “Hyperbolic Gap Material”, which is available in OpenSEES. This element has been assigned in each peripheral beam-column joint node and each middle node of the surrounded beam on the first floor. The static force–displacement relation of the HGM material and a schematic diagram of the location of the element is shown in Figure 9b,c. The surrounded soil has been designed in such a way as to keep the base isolator response within permissible limits.

## 4. Analytical Design Consideration of Building Frame

### 4.1. BRB Bracing System

**Figure 10:**
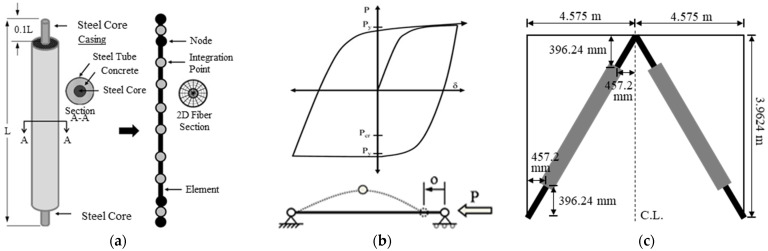
Buckling Restrained Braces System (BRBs): (**a**) BRB formulation; (**b**) BRB cyclic behavior; (**c**) Chevron bracing.

To include the bracing system in numerical analysis, a Buckling Restrained Braces System (BRBs) has been used, as shown in Figure 10. A steel core has been placed inside of a steel casing, and the gap has been filled with concrete to prevent local buckling of the steel core section and global buckling of a whole bracing member, as shown in Figure 10a. Composition of the fiber section and element formulation along with the material configuration is shown in Figure 10a [7]. The behavior under cyclic axial loading of the bracing is shown in Figure 10b, and the orientation of the bracing is shown in Figure 10c. Due to its ductile and high energy dissipation capacity, over the past few decades, engineers have started to use this bracing system. This bracing system also provides stable symmetric hysteretic behavior and high energy-dissipating capacity under inelastic conditions. Details of the physical dimensions and considered material property of the Buckling Re strained Bracing system (BRBs) are included in Table 3.

### 4.2. Design of Three-Dimensional Building Frame

**Figure 11:**
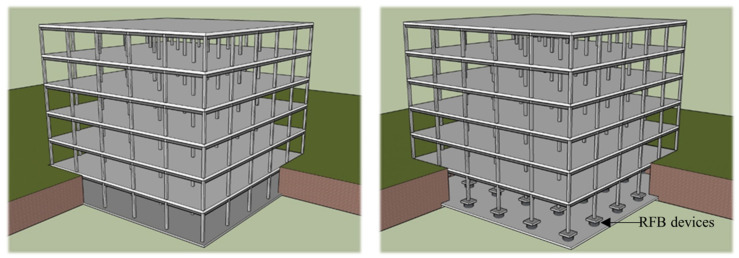
Three-dimensional view of unbraced simple frame model.

**Figure 12:**
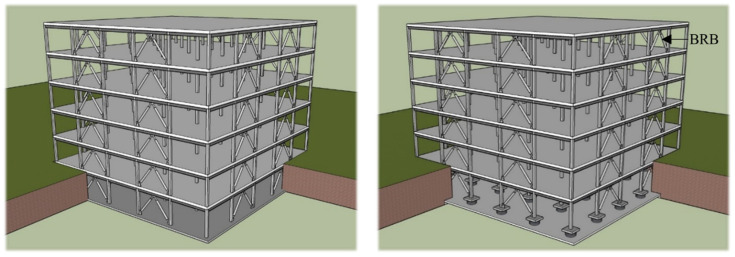
Three-dimensional view of braced frame model.

**Figure 13:**
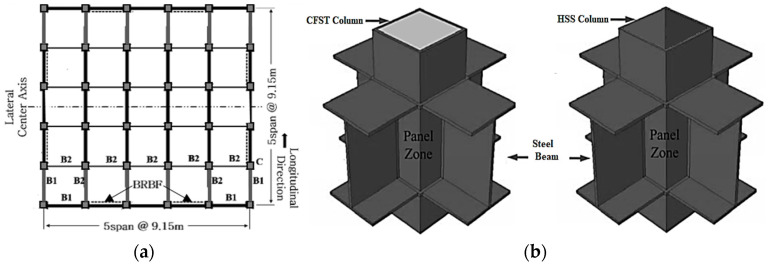
Building frame and beam–column connection system: (**a**) Frame plan view; (**b**) Beam–Column weld connection (CFST and HSS).

A well-established [7,56] three-dimensional six-story stable steel building frame has been selected, which has been designed according to ASCE 7-16 [26] guidelines to perform the numerical analysis. The base story has been considered as a basement. The simple frame and Buckling Restrained Braces Frame (BRBF) system are shown in Figure 11 and Figure 12 respectively. The load combination and structural members were designed in accordance with the AISC-LRFD manual [27]. The connection between the members has been considered as a welded connection to create a stiffness effect in the simulation process where rigid offset between members has been used. The structure has been considered as an ordinary office building which has seismic design category D surrounded by stiff well-graded silt sand and seismic hazard of 2% probability of exceedance in 50 years. The building was designed under the same and regular conditions without in-plane torsional effects due to the symmetrical plane in which mass and stiffness were uniformly distributed. Therefore, the height of each floor of the building is 3.9624 m and the bay is 9.15 m, which is uniform and symmetrical, as shown in Figure 13a. As indicated by dotted lines in the figure, two BRBF zones were installed on four sides. The beam–column joint of the frame indicated by thick lines in the plan view was designed with complete restraint. A uniform column section has been used in the design, while a relatively smaller beam section has been assigned to the higher floors. Among the various types of central braced frame systems, the widely used inverted V-type braced frame system was applied due to the advantages of architectural planning. In addition, in this study, two different column systems, CFST and HSS, were applied to the structure to evaluate the seismic performance according to the change of the column cross-section, as shown in Figure 13b. Basic consideration for designed dead loads and live loads is summarized in Table 4 where 1.2 DL + 1.0 LL combination has been utilized. Lumped mass and load have been assigned to the main nodes. The beam column detail member size has been summarized in Table 5. All frame members have been designed as a nonlinear beam–columns with 3D fiber sections. To incorporate the force–displacement behavior of RFB devise equivalent, a Lead Rubber Bearing (LRB) has been used with previously defined material property. The recorder command that is available in OpenSEES has been used to collect all data.

## 5. Designed Ground Motion

**Figure 14:**
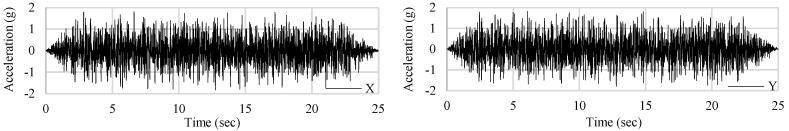
Representative strong (1.8 g) artificial earthquake.

To perform the nonlinear dynamic analysis, two sets of eleven bi-directional artificial earthquakes data have been used designed by PRISM software [57], one set consisting of six moderate (PGAs 0.5 g) earthquakes and another containing five strong (PGAs 1.8 g) earthquakes. Moderate earthquake data have been used to calculate the base isolator response, which was discussed in Section 2.3. All artificial strong ground motion has a similar duration of 25 s in both directions. Artificial single strong ground motion data are shown in Figure 14.

**Figure 15:**
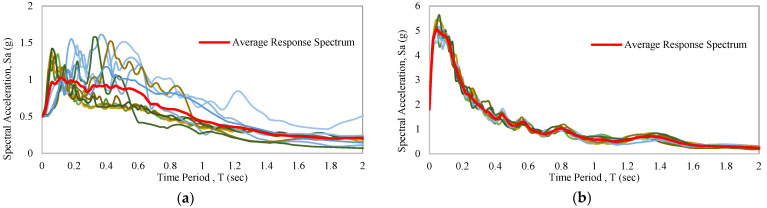
Average response spectrum of considered EQs: (**a**) 0.5 g earthquakes average response spectrum; (**b**) 1.8 g earthquakes average response spectrum.

**Figure 16:**
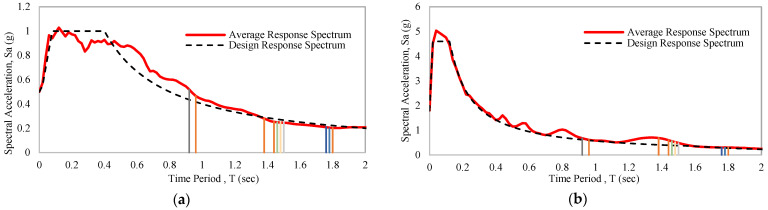
5% Damped probability of exceedance 2% in 50 years designed response spectrum of considered EQs: (**a**) 0.5 g earthquakes design response spectrum; (**b**) 1.8 g earthquakes design response spectrum.

A seismic response spectrum is commonly used for nonlinear dynamic analysis. The maximum value of the response spectrum can be displacement, velocity, or acceleration. The average response spectrum of the following earthquake data is shown in Figure 15a,b. Details of the considered variables to design the response spectrums have been discussed for 0.5 g and 1.8 g earthquake, respectively, where 5% damped probability of exceedance 2% in 50 years has been considered [58]. The Maximum Considered Earthquake (MCER) spectral response (SS) coefficients for short periods of 0.2 s are 1.5 and 6.9, while values of S1 for a period of 1 s are 0.24 and 0.34. Local amplification factors for short periods (Fa) and long periods (FV) are 1 and 1.6. Spectral acceleration values at short periods (SMS) adjusted for site class effects of MCER are 1.5 and 6.9; for a period of 1 s (SM1); these values are 0.384 and 0.544. Design spectral acceleration parameters at short periods (SDS) are 1 and 4.6; for a period of 1 s (SD1), these values are 0.256 and 0.36267. The initial period (T0) values are 0.0512 and 0.01577. The short-period transition (TS) values for small structures are 0.256 and 0.07884. The long-period transitions for target structures are 2.62 and 1.6. The design response spectrum of moderate and strong artificial earthquakes is shown in Figure 16a,b.

## 6. Response Spectrum Analysis

For each dominant natural period (T) of different frame models, the average response spectrum acceleration is given in Table 6, where Sa * and Sa ** define spectral acceleration under 0.5 g and 1.8 g earthquakes, respectively.

A simple frame with a hollow structural section has a slightly higher natural period of 1.440 s compared to a concrete-filled tube column frame, which has 1.393 s. The corresponding spectra acceleration of the CFST and HSS frame are 0.280 g and 0.253 g under a 0.5 g earthquakes design response spectrum. For a 1.8 g earthquakes design response spectrum, these values are 0.690 g and 0.598 g. If a bracing system is present in the building frame, the natural period decreases to 0.963 and 0.936 s, respectively. The average spectral acceleration under braced conditions becomes 0.520 g and 0.466 g for 0.5 g earthquakes. In 1.8 g earthquakes, these values are 0.680 g and 0.610 g. In the presence of a base isolator regarding the base isolator types, a simple frame shows a high natural period between a range of 1.767 of 1.819 s compared to a braced frame’s range of 1.478–1.520 s. A base isolated simple frame exhibits around 0.2 g and 0.3 g under 0.5 g and 1.8 g spectral acceleration. For base-isolated braced frames, these values increase slightly to 0.25 g and 0.48–0.56 g.

## 7. Nonlinear Time History Analysis (NLTHAs)

In this study, all prototype building frames have been investigated through NLTHAs by using eleven earthquakes with a 2% probability of exceedance in 50 years. A static step has been used to apply the initial load over the frame model after which dynamic analysis has been applied. A transformation constraint, sparse general system, and Newton Line Search Algorithm with stable tolerance and default iterations have been used. An energy increment convergence test has been used in both stages.

### 7.1. Peak and Residual ISDR

The section shows the maximum average displacement response and residual response of the frames under two sets of earthquakes. Subsequent figures show the average maximum ISDR of a simple frame without any base isolation system and with a base isolation system that has two different column systems. A BRB braced frame response has been shown depending on the column and base isolation presented in the analysis.

**Figure 17:**
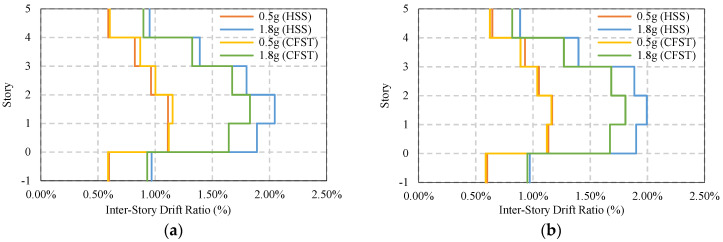
Average maximum story drift of simple frame under EQs: (**a**) Simple frame average max ISDR at X−direction; (**b**) Simple frame average max ISDR at Y−direction.

Figure 17 shows maximum average story drift of simple frames without considering any bracing and base isolator. It has been found that under moderate ground motion, both the HSS and CFST frame shows similar story drift, approximately 1.12%, but in the presence of a strong seismic event, the HSS frame shows higher story drift compared to the CFST frame, which marginally fulfills the design limit of 2% [59,60]. The CFST frame shows safer behavior compared to the HSS frame. The majority of the drift appears on the second floor, as shown in Figure 17a,b.

**Figure 18:**
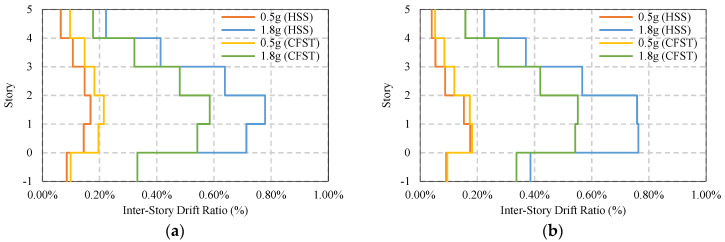
Average maximum residual story drift of a simple frame under EQs: (**a**) Simple frame average residual ISDR in the X−direction; (**b**) Simple frame average residual ISDR in the Y−direction.

The average maximum residual story drift of the same frame system is shown in Figure 18a,b where it indicates the permanent deformation of the frame after an earthquake. Under moderate ground motion, the damage in both HSS and CFST frames is similar, which is around 0.2% story drift. However, in a strong earthquake, the HSS frame has higher damage compared to the CFST frame, which is almost 0.80% and 0.60%, respectively. Most of the damage accumulated in the first and second story of the building frame. Due to the presence of soil around the basement, the story drift of the frames at the basement level does not exceed 0.4% under strong ground motion.

**Figure 19:**
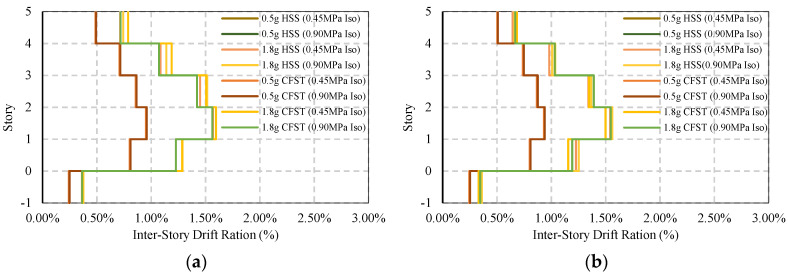
Average maximum story-drift of base isolated simple frame under EQs: (**a**) Simple frame average max ISDR in the X−direction; (**b**) Simple frame average max ISDR in the Y−direction.

The average maximum response of base-isolated simple frames is shown in Figure 19a,b where both frames show controlled responses. The presence of a base isolator shows a safer response of the frame under strong ground motion compared to the moderate earthquakes. Under moderate earthquakes, the story drift reduces down to 0.95% from 1.15%. In strong ground motion, this value is 1.56%, where without base isolation, this value is almost 2%.

**Figure 20:**
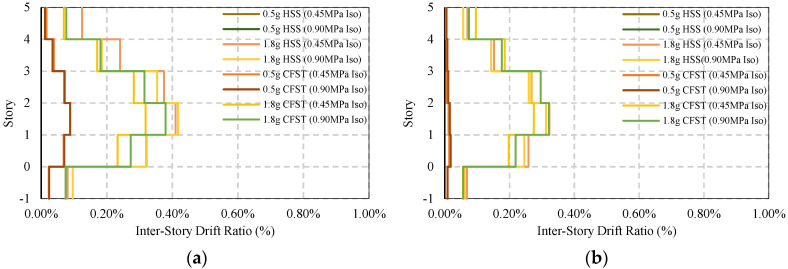
Average maximum residual story drift of base isolated simple frame under EQs: (**a**) Simple frame average residual ISDR in the X−direction; (**b**) Simple frame average residual ISDR in the Y−direction.

The residual drift of the frame is given in Figure 20a,b where the base isolator shows significant damage reduction regardless of the ground motions. Under moderate earthquakes, the damage reduced from 0.20% to 0.08% and 0.02% in the X and Y directions, correspondingly. Under strong earthquakes, the damage is reduced to 0.40% and 0.32% in the X and Y directions.

**Figure 21:**
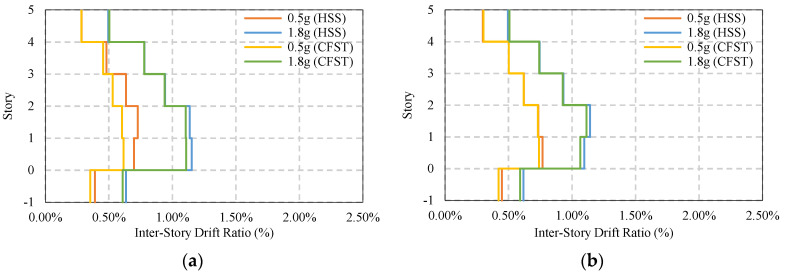
Average maximum story drift of BRBs frame under EQs: (**a**) BRBs frame average max ISDR in the X−direction; (**b**) BRBs frame average max ISDR in the Y−direction.

**Figure 22:**
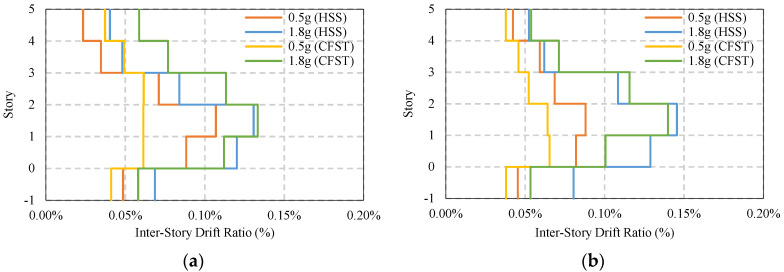
Average maximum residual story drift of BRBs frame under EQs: (**a**) BRBs frame average residual ISDR in the X−direction; (**b**) BRBs frame average residual ISDR in the Y−direction.

Figure 21 and Figure 22 present the response of braced frame (BRBs) having two different column systems under moderate and strong earthquake loads where no base isolator has been used. The maximum story drift under 0.5g earthquakes is 0.76%, and that under 1.8g earthquakes is 1.15%. The maximum post-earthquake damage is 0.14% under both moderate and strong ground motions, which are considered to be safe in design standard limit 0.20%. In addition, this value is much lower compared to the base-isolated simple frame conditions.

**Figure 23:**
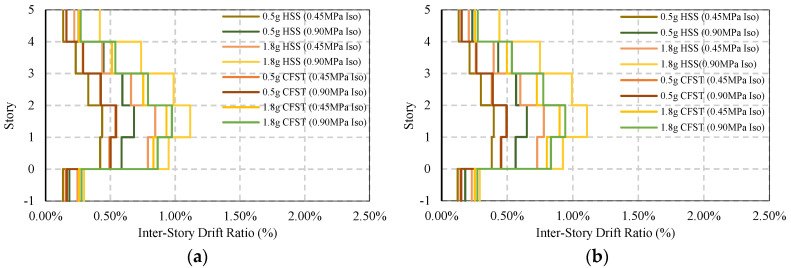
Average maximum story drift of BRBs base isolated frame under EQs: (**a**) BRBs frame average max ISDR in the X−direction; (**b**) BRBs frame average max ISDR in the Y−direction.

**Figure 24:**
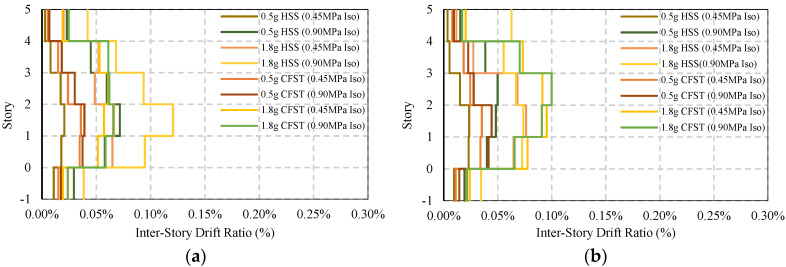
Average maximum residual story drift of BRBs base isolated frame under EQs; (**a**) BRBs frame average residual ISDR in the X−direction; (**b**) BRBs frame average residual ISDR in the Y−direction.

The maximum story drift response and residual response of a base-isolated braced frame are shown in Figure 23 and Figure 24. For an HSS column, the response shows some variation depending on the direction of the earthquake, whereas in the presence of a CFST column, the response is similar. The damage is almost 0.12% to 0.1%, as shown in Figure 24a,b under strong ground motions.

**Figure 25:**
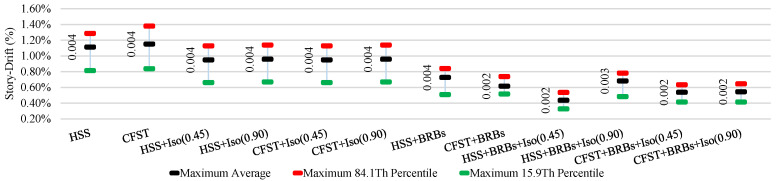
Inter-story drift ration of different cases in the X-direction under moderate EQs.

**Figure 26:**
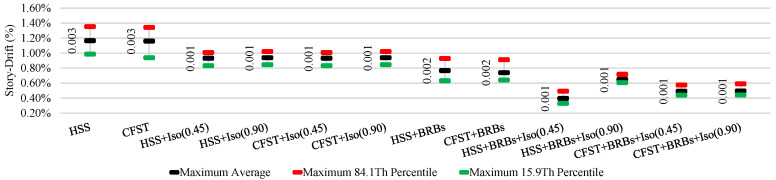
Inter-story drift ration of different cases in the Y-direction under moderate EQs.

**Figure 27:**
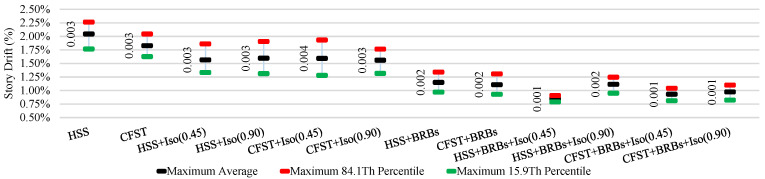
Inter-story drift ration of different cases in the X-direction under strong EQs.

**Figure 28:**
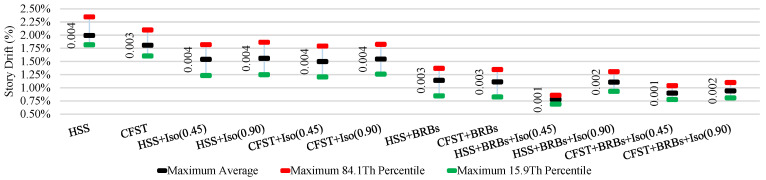
Inter-story drift ration of different cases in Y-direction under strong EQs.

The average ISDR of different frames in different directions under moderate and strong earthquakes is shown in Figure 25, Figure 26, Figure 27 and Figure 28. The vertical axis value represents the percentage of story drift and in the horizontal direction, and twelve different cases have been shown. Values in the figures show the standard deviation of the corresponding dataset. Red, black, and green lines denotes the maximum 84.1th percentile, average, and 15.9th percentile values, respectively. From Figure 25, Figure 26, Figure 27 and Figure 28, the standard deviation value is lower, which means that the dataset is clustered around the mean value and highly reliable. From the stock diagrams, it has been found that bracing systems provide significant control on an embedded frame response over base isolator systems. The presence of bracing and a base isolator provides a superior controlled response along with minimal damage to the structure.

**Figure 29:**
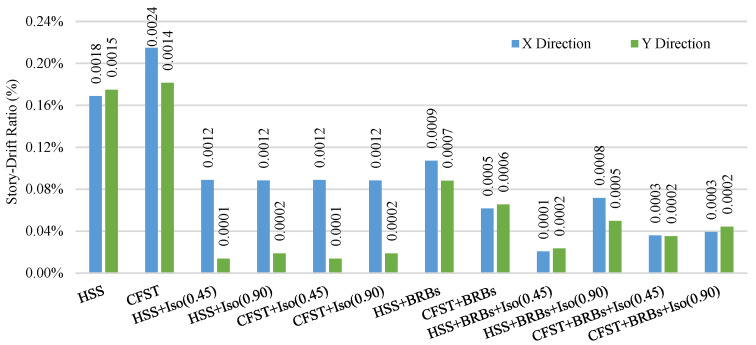
Average residual story drift under moderate (0.5 g) EQs.

**Figure 30:**
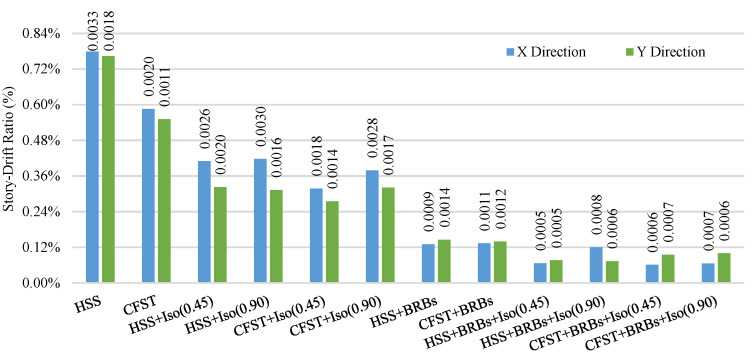
Average residual story drift under strong (1.8 g) EQs.

Figure 29 and Figure 30 show the average residual story drift of different cases under moderate and strong earthquakes, respectively, where data corresponding to the residual story drift shows the standard deviation of the datasets. Diagrams show that isolators significantly reduce the damage under moderate earthquakes relative to strong ground motions. Bracing systems provide more damage reduction for embedded frame systems under strong earthquakes. The presence of bracing and a base isolator provides greater safety in the presence of moderate and strong ground motions.

### 7.2. Soil Spring Response

Soil spring reaches approximately 825 kN lateral pressure under moderate earthquakes regardless of column types, which reaches around 1200 kN under strong earthquakes. All base-isolated simple frames consisting of HSS and CFST columns have shown the same response: about 2000 kN under moderate and 2500 kN under strong earthquakes. The maximum passive force of the non-isolated braced frame building reaches 750 kN from 650 kN. This reduction occurs due to the presence of BRBs, which reduces the lateral story drift in the building frame. Under moderate earthquakes, the base-isolated braced frame experiences almost 1700 kN lateral pressure, which reaches 2675 kN in strong ground motions.

**Figure 31:**
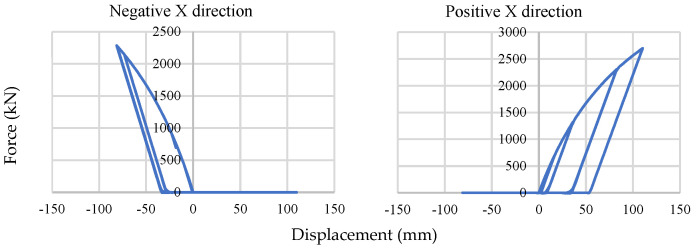

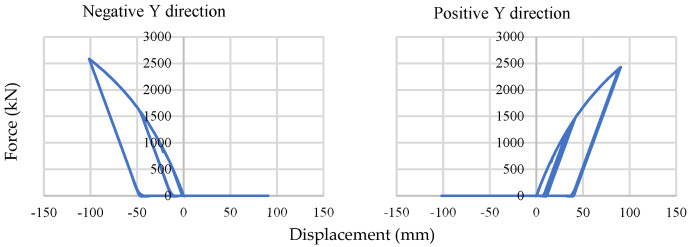
Single soil spring response.

Figure 31 shows comparatively high force and displacement under base-isolated braced frame conditions, and it reaches almost 2675 kN in one direction. In all cases, the displacement is within ±150 mm.

### 7.3. Base Isolator Response

The base isolator behavior under bi-directional earthquakes is presented in this section. Under moderate and strong earthquakes, the response of each isolator is within the permissible limit, which is ±150 mm in both directions. The base isolator under CFST and HSS unbraced and braced frame systems shows similar performance depending on the specific base isolator (0.45 MPa and 0.90 MPa) property and earthquake’s intensity.

**Figure 32:**
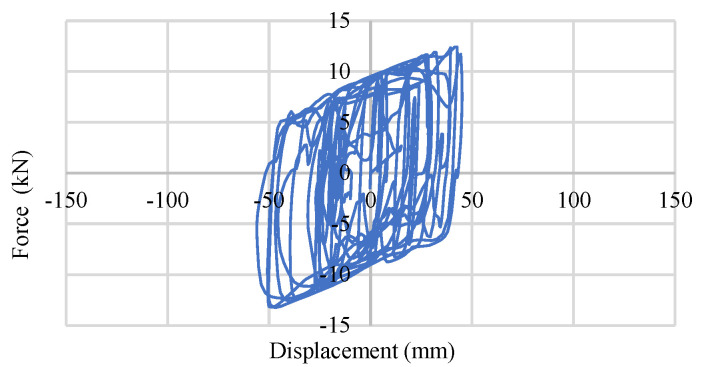
Single LRB-0.45 MPa response under moderate EQ.

**Figure 33:**
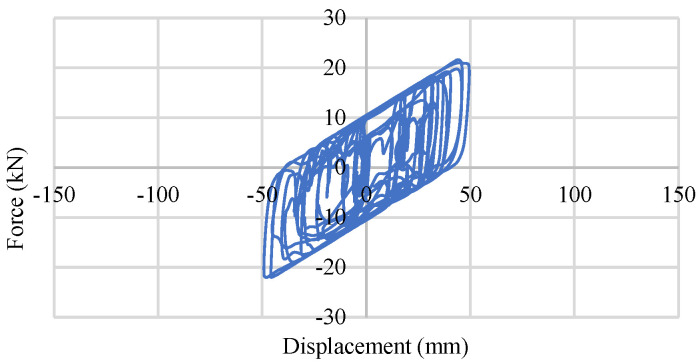
Single LRB-0.90 MPa response under moderate EQ.

**Figure 34:**
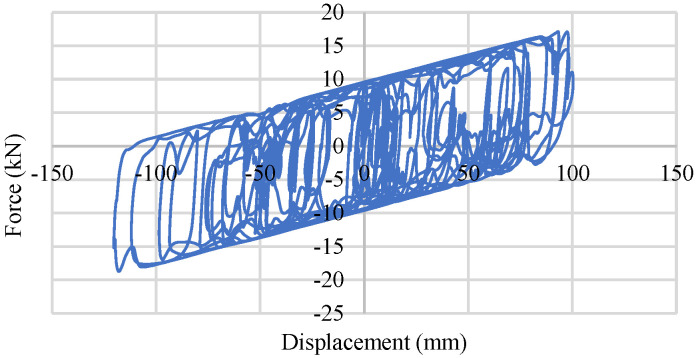
Single LRB-0.45 MPa response under strong EQ.

**Figure 35:**
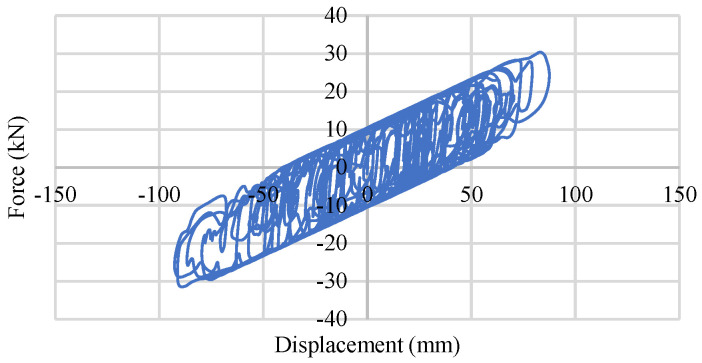
Single LRB-0.90 MPa response under strong EQ.

In Figure 32 and Figure 33, the response of a 0.45 MPa and 0.90 MPa base isolator under the BRBs–CFST frame is shown. This behavior generates under moderate earthquakes where the maximum displacement is approximately 50 mm. Figure 34 and Figure 35 show the base isolator response under the strong bi-directional ground motions of the same frame system where the maximum values are approximately 120 mm and 92 mm for 0.45 MPa and 0.90 Mpa, respectively. The rest of the models show similar behavior.

### 7.4. Shear Force vs. Roof Displacement

A non-isolated simple building frame shows around 15 MN and 25 MN base shear force under moderate and strong earthquakes, respectively. The base shear reduces to 0.45 to 0.5 MN under moderate ground motion, regardless of the isolation system. The maximum base shear reduces down to 0.6 MN at the time of high-intensity earthquakes, which is 1.8 g, under which both isolation systems provide similar base shear reduction. The base shear response is almost similar under bracing conditions and different column systems.

**Figure 36:**
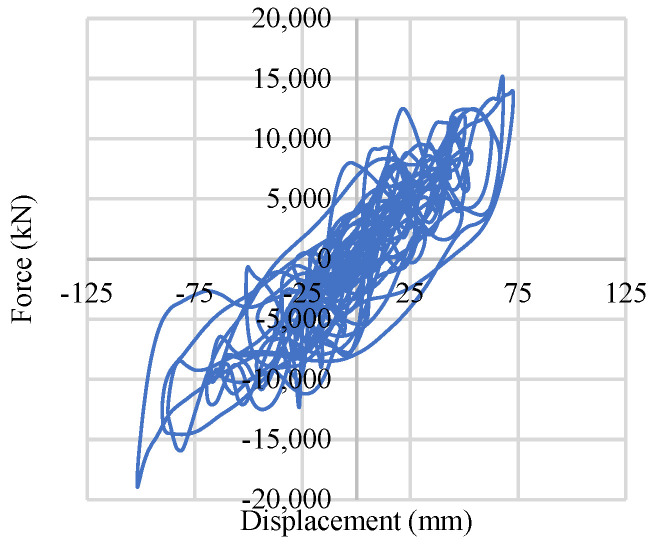
Non-isolated frame shear force vs. roof displacement under 0.5 g EQs.

**Figure 37:**
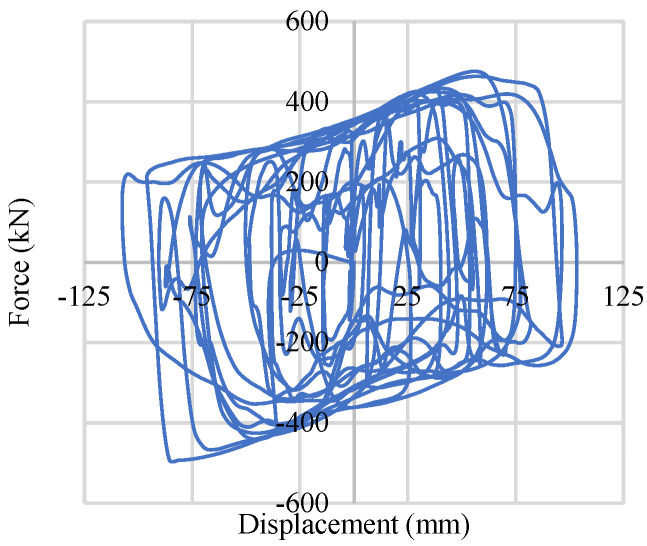
Isolated 0.45 MPa frame shear force vs. roof displacement under 0.5 g EQs.

**Figure 38:**
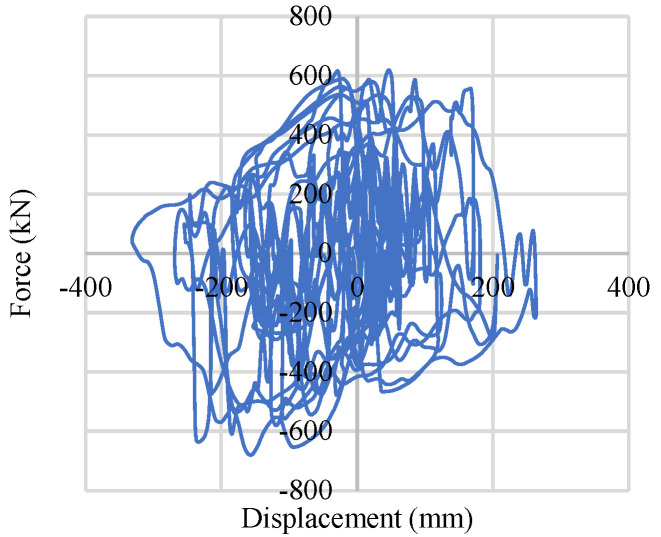
Isolated 0.45 MPa frame shear force vs. roof displacement under 1.8 g EQs.

The behaviors of non-isolated and isolated BRB-CFST frame systems have been shown in Figure 36, Figure 37 and Figure 38. Figure 36 shows the base shear response of a non-isolated BRB-CFST frame under a 0.5 g earthquake, where the maximum shear force is around 19 MN. This shear force reduces down to 0.5 MN under the same earthquake when a 0.45 MPa base isolator is present in the system, which is shown in Figure 37. Figure 38 shows the base shear value along with the roof displacement of an isolated BRB-CFST frame under strong ground motions. Under a 1.8 g earthquake, the base-isolated BRB-CFST frame system showed approximately 0.68 MN base shear.

## 8. Conclusions

In this study, the performance has been studied of two different column systems, HSS and CFST, which are used in an ordinary office building that is surrounded by stiff soil and located in a high-seismic metropolitan area. The same frame equipped with two different base isolation systems, Rubber Friction Bearing (RFB) and conventional Buckling-Restrained Braces (BRB) systems, has been used to see the controlled response, and a comparison has been made between the two regarding their improved performance. Overall, the performance under two different sets of seismic inputs (0.5 g and 1.8 g) has been investigated. The following conclusions can be drawn from the output results:

1. From the data of story drift, it is clear that due to the presence of the basement in the building system, the maximum story drift and post-earthquake damage is concentrated in the second floor of this six-story building frame.
2. Under moderate earthquakes, both the HSS and CFST frame behavior is similar, but the HSS frame shows some additional story drift compared to CFST. The average story drift response of both simple frames under moderate earthquakes is safer, which is below the 2% provided by ASCE limits, but under strong ground motion, the 84.1th percentile line crosses this limit. The residual drift of the simple frame shows a similar damage pattern under 0.5 g earthquakes. This damage in terms of residual story drift reaches 0.60% for the CFST frame and almost 0.80% for the HSS frame if strong ground motions are applied that are over the safety limit 0.50% of the Japanese standard.
3. The presence of RFB in both frames shows controlled behavior by showing 1% and 1.5% story drift under moderate and strong ground motion, respectively. The most significant improvement happens in damage control, where it reduces by 70% and 50% damage under moderate and strong ground motion individually. In a simple frame, the presence of base isolation controls story drifts at the basement that are below 0.5%, which exceeded 0.5% without base isolation condition.
4. Due to the surrounding stiff soil, the response and damage of the frame cannot be reduced significantly, even though base isolator system is present. To improve the performance, a few BRB have been implemented that show more improvement in the response by reducing the story drift by around 34% to 46% and damage by about 40% to 80%, respectively, depending on the intensity of the ground motions (0.5 g and 1.8 g) and direction.
5. The presence of a base isolator and bracing system in the frame shows superior results by reducing the story drift by 20% and residual story drift by around 40%. For the CFST frame, this reduction is stable, but for the HSS frame, this reduction is not stable in both directions. One direction shows higher reduction compared to the other directions in this three-dimensional frame building. Damage is also negligible, which is less than 0.15% in terms of residual story drift.
6. In the presence of a base isolator, a significant portion of base shear force is reduced when strong ground motion has been applied. The experimental results do not show any significant differences between those two different base isolator systems. In the simulation process, the results are also similar. The overall frame response improved under base isolation conditions.
7. From a performance point of view, the HSS frame equipped with a base isolator system can be a good alternative for the CFST frame system when moderate earthquakes are dominant. The base isolator cannot itself reduce the damage of the superstructure due to the presence of the surrounding soil in a frequent strong seismic zone. For embedded frames, the combination of base isolator and minimum bracing can be a good choice to safeguard the structure and minimize the damage. Therefore, these systems can not only reduce property damage caused by earthquakes but also reduce the maintenance costs caused by damage to structures due to their excellent seismic performance.

## 9. Limitations and Future Studies

This study has been limited by the simulation results where it has not been possible to perform the real-world three-dimensional frame experiment due to the absence of proper matter, equipment/systems, and funds. The backbone curve of soil that has been used was taken from another study. For the superstructure, the joint has been considered welded, and the earthquakes that have been considered are artificial ground motions, which are also some limitations of this study.

Future studies can be conducted to develop RFB elements in the software platform. Designing proper joints for this three-dimensional superstructure and evaluating the response can be a good point for research. Field experiment and simulating backbone curves for different soil types can be a novel area of investigation. Response evaluation of symmetric and asymmetric buildings under designed earthquakes can also be a new research topic. In addition, in this study, there are research limitations on high-strength concrete, which is applied to most structures today. Therefore, additional seismic and fire-resistance performance evaluation studies for structures to which high-strength concrete is applied and optimization studies using various algorithms for structures to which RFB is applied are planned.

## Figures and Tables

**Table 1:** Mechanical properties of the RFB.

Symbols	0.45 MPa Design Value	0.90 MPa Design Value
Fmax	12.4 kN	20.52 kN
Qd	8.96 kN	9.86 kN
k2	0.081 kN/mm	0.23 kN/mm
keff	0.248 kN/mm	0.38 kN/mm
EDC	1769.31 kN/mm	1945.65 kN/mm

**Table 2:** Design properties of “LeadRubberX”.

Symbols	0.45 MPa Design Value	0.90 MPa Design Value
Yield Strength (Fy)	9.5 kN	10.5 kN
Post-Yield Stiffness Ratio (α)	0.010	0.030
Shear Modulus (Gr)	0.250 MPa	0.950 MPa
Bulk Modulus of Rubber (Kbulk)	1.344 MPa	2.6887 MPa
Internal Diameter (D1)	370 mm	370 mm
Outer Diameter (D2)	435 mm	435 mm
Steel Shim Thickness (ts)	20 mm	20 mm
Rubber Layer Thickness (tr)	10 mm	10 mm
Number of rubber layers (n)	19	19

**Table 3:** Physical and material property of Buckling Restrained Braces System (BRBs).

Section (OpenSEES Material)	Length	Diameter (d) or Thickness (t)	Fy (MPa) or f_pc_ (MPa)	E_0_ (MPa) or f_pcu_ (MPa)	b	ε_psc0_ (mm/mm)	ε_psu_ (mm/mm)
Steel Core (Steel01)	6.05 m	57.15 mm (d) up to 4th story 50.80 mm (d) 5th & 6th story	345	200,000	0.01	-	-
Steel Tube (Steel01)	4.84 m	6.35 mm (t)	290	200,000	0.01	-	-
Concrete (Concrete01)	4.84 m	41.275 mm (t)	−34.5	−27.579	-	−0.003	−0.06

**Table 4:** Basic consideration for building design (DL = dead load, LL = live load).

Located Area	Loads (Other)	Loads (Roof)	Coefficient	Site Condition	Category
High seismic zone having stiff well-graded silt sand	DL: 4.12 kN/mm^2^ LL: 2.39 kN/mm^2^	DL: 4.05 kN/mm^2^ LL: 0.96 kN/mm^2^	DL: 1.2 LL: 1.0	Stiff Soil	Ordinary structure

**Table 5:** Member property of prototype frame building.

Story	Column ^a^	Peripheral Beam ^a^ (B1)	Internal Beam ^a^ (B2)	BRBs	
				Core Area ^a^ (mm^2^)	Casing Tube ^b^
1	HSS18 × 18 × 7/8	W24 × 84	W24 × 68	2580	HSS6 × 1/4
2	HSS18 × 18 × 7/8	W24 × 84	W24 × 68	2580	HSS6 × 1/4
3	HSS18 × 18 × 7/8	W24 × 68	W24 × 68	2580	HSS6 × 1/4
4	HSS18 × 18 × 7/8	W24 × 68	W24 × 68	2580	HSS6 × 1/4
5	HSS18 × 18 × 7/8	W18 × 50	W24 × 68	2027	HSS6 × 1/4
6	HSS18 × 18 × 7/8	W18 × 50	W24 × 68	2027	HSS6 × 1/4

^a^: Gr50 Carbon Steel, ^b^: GrB Carbon Steel.

**Table 6:** Average spectral acceleration corresponding to the fundamental period (T).

Prototype Building Configuration		Concrete-Filled Steel Tube (CFST) Column			Hollow Structural Sections (HSS) Column		
		T (second)	Sa * (g)	Sa ** (g)	T (second)	Sa * (g)	Sa ** (g)
Simple Frame Building		1.393	0.280	0.690	1.440	0.253	0.598
Base Isolated Simple Frame Building	0.45 MPa Iso.	1.767	0.210	0.300	1.804	0.202	0.303
	0.90 MPa Iso.	1.781	0.200	0.300	1.819	0.202	0.303
Braced Frame Building		0.936	0.520	0.680	0.963	0.466	0.610
Base Isolated Braced Frame Building	0.45 MPa Iso.	1.478	0.250	0.560	1.501	0.250	0.480
	0.90 MPa Iso.	1.497	0.250	0.520	1.520	0.250	0.480

* Sa: Spectral acceleration under moderate EQs spectrum; ** Sa: Spectral acceleration under strong EQs spectrum.

## Data Availability

Not applicable.
